# Bilateral ossification of the auricles: an unusual entity and review of the literature

**DOI:** 10.1186/1746-160X-5-17

**Published:** 2009-10-01

**Authors:** Nicholas S Mastronikolis, Peter Zampakis, Christina Kalogeropoulou, Theodoros Stathas, Vassiliki Siabi, Eleni Geropoulou, Panos D Goumas

**Affiliations:** 1 Department of Otorhinolaryngology-Head and Neck Surgery, University Hospital of Patras, Patras, Greece; 2 Department of Radiology, University Hospital of Patras, Patras, Greece; 3 Department of Internal Medicine, University Hospital of Patras, Patras, Greece; 4 Department of Pathology, University Hospital of Patras, Patras, Greece

## Abstract

**Background:**

True ossification of the auricle with cartilage replacement by bone, is a very rare clinical entity and can result in an entirely rigid auricle.

**Case presentation:**

We present a rare case of bilateral ossification of the auricles in a 75-years old man with profound progressive rigidity of both auricles. His main complaint was a mild discomfort during resting making sleeping unpleasant without any other serious symptoms. His medical history was significant for predisposing factors for this condition such as, Addison's disease and diabetes mellitus. Excisional biopsy was performed confirming the ossified nature of the auricles. Further treatment deemed unnecessary in our case due to his mild clinical picture.

**Conclusion:**

True auricular ossification is a quite rare clinical entity with unclear pathogenesis and one should have in mind that there is always the possibility of a serious co-existed disease like endocrinopathy.

## Introduction

True ossification or petrification of the auricle with cartilage replacement by bone, resulting in a stiff auricle, is a very rare clinical entity.

The commonest cause of "stiff auricle" condition, is ectopic calcification, rather than ossification [1]. Bochdalek in Prague (1866) [2] first described a case of bilateral ear calcification with histological confirmation, while Wassmund [3] in 1899 first reported the X-ray findings of this condition. Since then, many cases of rigid ears have been reported in the literature usually as a result of calcification [4], but true ossification with ectopic bone formation has been histologically documented in only 17 previous cases [1,4-18].

Local trauma [12,17], frostbite [1,4,11], inflammations [16,18], and various systemic diseases and endocrinopathies [11,19-22] have been considered as causative factors, however, this condition can also occur without any known predisposing cause or event [1,5,17].

We present the 18^th ^pathologically documented case of bilateral auricular ossification and review the relevant literature.

## Case report

A 75-year-old man presented in our clinic with a history of a progressive stiffness of both auricles over the last 15 years. He was referred to us from the Department of Internal Medicine of our Hospital for further evaluation and due to his complaints for a mild discomfort when resting in each side thus making sleeping unpleasant. He also observed a gradual hearing loss on both ears along with tinnitus and intermittent blockage, especially on the left ear.

**Figure 1:**
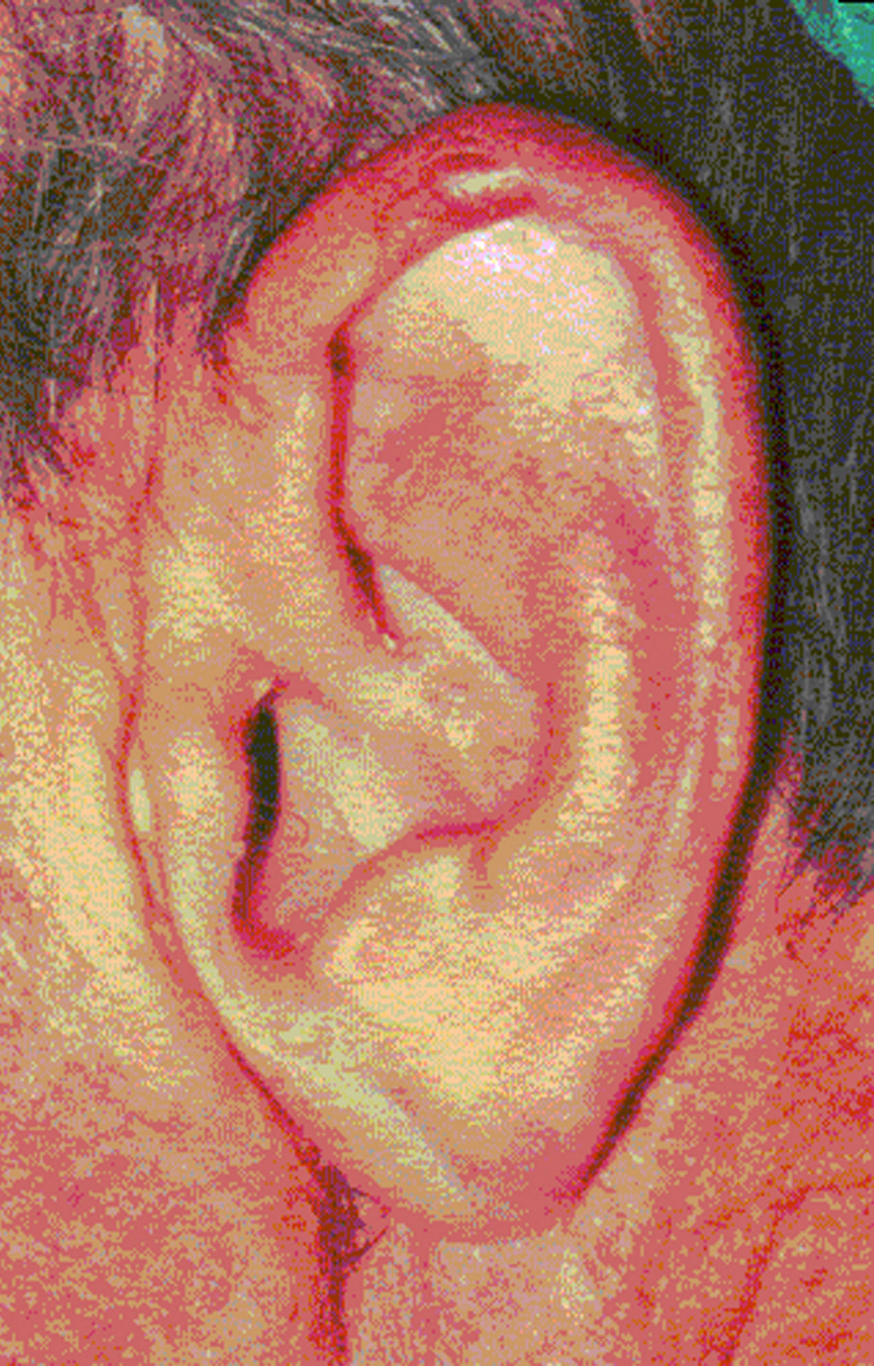
**Normal auricle configuration and skin appearance - nodule on the top of the helix**.

Routine ENT examination demonstrated a profound bilateral rigidity of both auricles, which were completely inflexible in their entirety except of the lobules, moving as a single unit with manipulation. Lesions resembling chondrodermatitis nodularis chronicus helicis was coexistent in both auricles with typical raised and firm nodules but without tenderness. The rest of the auricles configuration and skin appearance were normal without noticeable cutaneous abnormalities. Some thickening and hardening was noticed on palpation (Figure. 1). There was no sensation deficit. The ear canals were free of cerumen and wide enough for a thorough otoscopy. The right eardrum appeared normal, while the left one had an extensive tympanosclerotic plaque on its lower half. Palpation of nasal, thyroid and cricoid cartilages did not reveal any abnormalities and the epiglottis and arytenoids had a normal appearance on laryngoscopy. The rest of the ENT examination was without any significant abnormal findings. A scar from a previous superficial parotidectomy was visible in the right side. The patient denied frostbite, wrestling, boxing or any other activity could provoke local injury, physical trauma or inflammation to his ears. He only mentioned some episodes of otitis media in his left ear during early childhood without serious sequelae. The patient could not recall any familial occurrence of auricular rigidity. His medical history was significant for heart and renal failure, Addison's disease, diabetes mellitus and epilepsy episodes. A thorough laboratory evaluation was performed including complete blood count and biochemistry profile, as well as thyroid function tests and parathyroid hormone levels measurement. Except of a mild elevation on glucose levels, the rest results were within normal limits.

**Figure 2:**
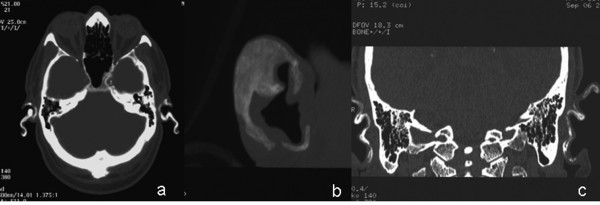
**CT images**. a. Axial CT images demonstrate ossification along both auricles. b. 3D images: complete ossification of both auricles. c. Tiny radiolucent areas within bony opacities are better seen in coronal reformatted images, indicating true bone formation.

The patient underwent a CT scan of the skull in order to investigate the cause of the auricular stiffness. Axial CT images demonstrated bilateral high-density opacities along the auricles consistent with ossification and true bone formation in both auricular cartilages. A very strong indicative factor for ossification and true bone formation along the auricles, was the presence of radiolucent areas within the dense opacities^8 ^(Figures 2a, b, c). Furthermore these axial CT images were also reconstructed with a thin slice bony algorithm and coronal, sagittal and 3D reformatted images obtained. The full extend of the ossifications was demonstrated. Chest X-ray was normal, without calcification of the costal cartilages.

A pure tone audiometry showed a moderate sensorineural hearing loss in the right ear (50 db at 1 kHz) and a severe mixed type hearing loss in the left ear (80 db at 1 kHz), Tympanometry was normal in both ears.

**Figure 3:**
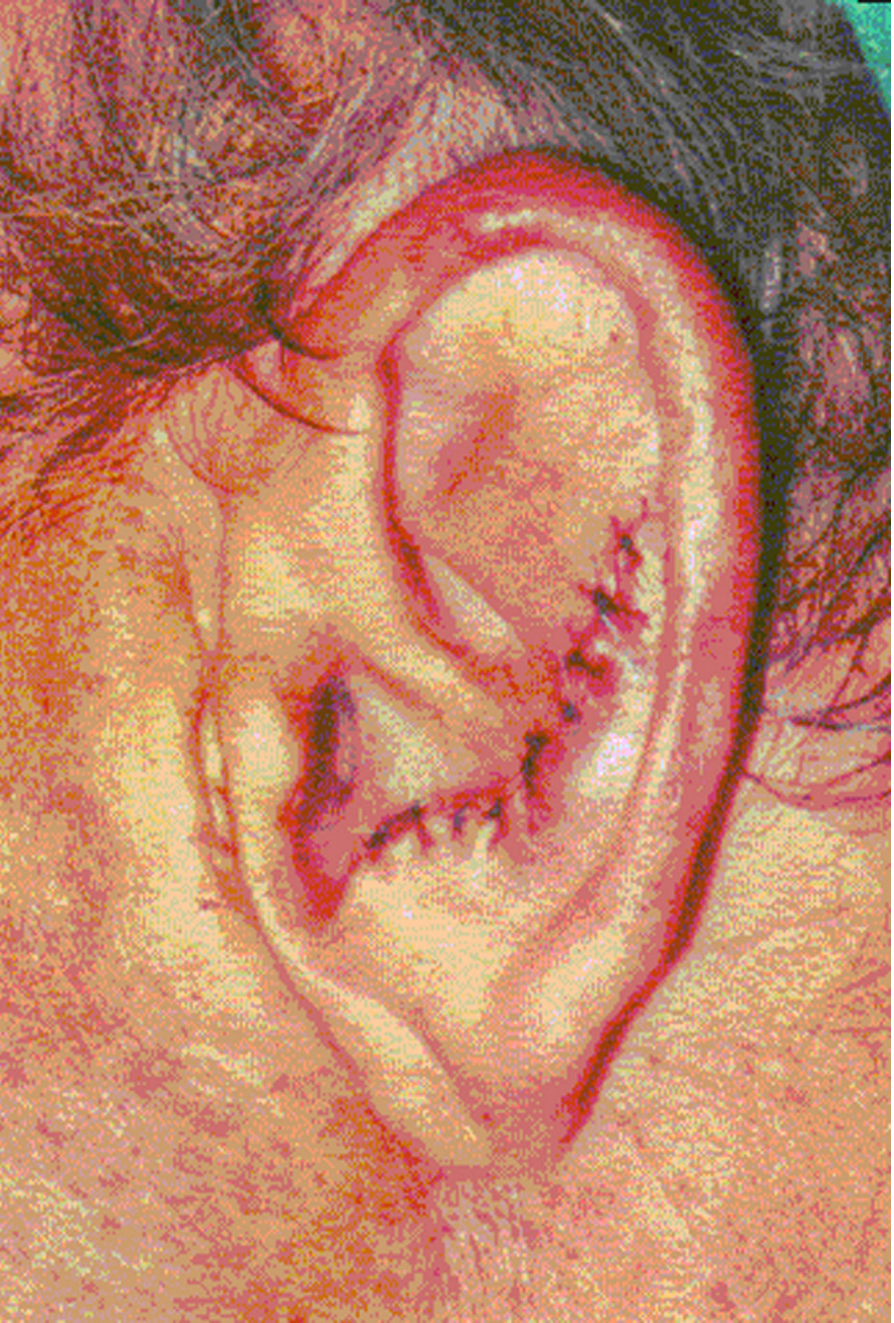
**Site of excisional biopsy for histological evaluation**.

Consequently, the patient underwent an excisional biopsy from the left ear for histological evaluation (Figure 3). Because of the absence of certain tenderness or pain, the patient did not agreed for an extensive auricular surgery.

**Figure 4:**
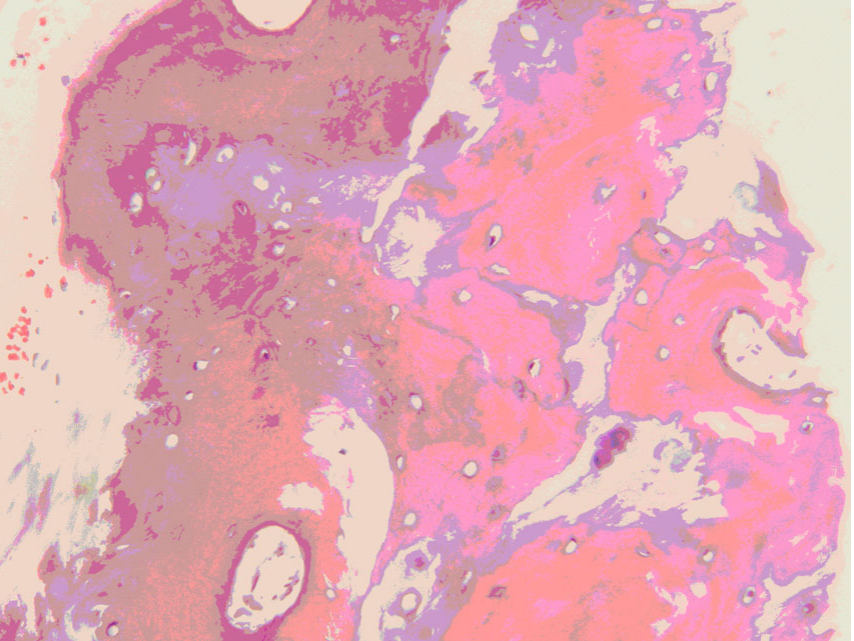
**Normal bone structure showing Haversian canals, cement lines and osteocytes**.

**Figure 5:**
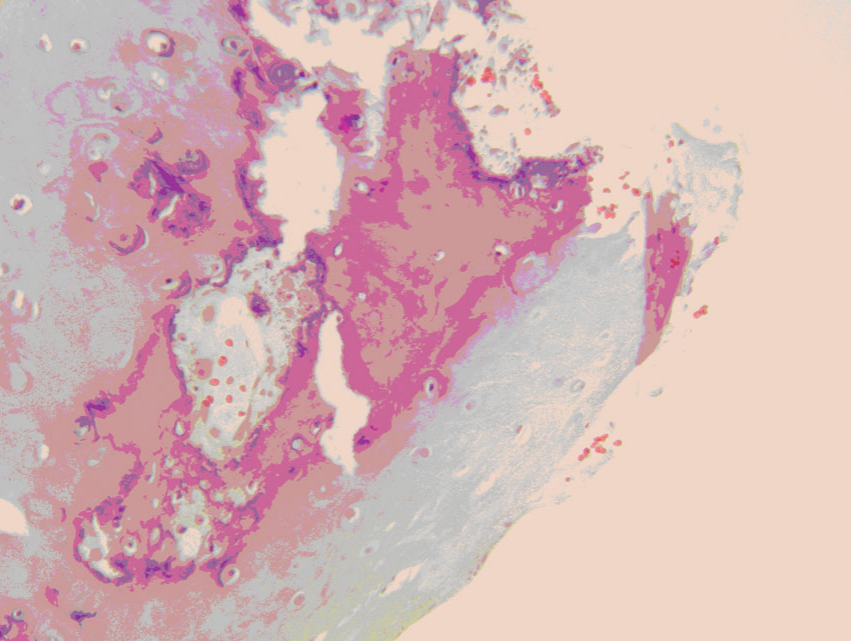
**Fragment of elastic cartilage with focus of mature bone formation**.

Histological sections of the biopsy specimen revealed spicules of lamellar bone with cement lines and Haversian canals, osteocytes, stromal component of adipose tissue, and fragments of elastic cartilage. Osteoblastic activity was not prominent (Figure 4 and Figure 5).

## Discussion

The auricle is consisted mainly of auricular cartilage, a type of elastic cartilage containing numerous elastic fibers. Except of the auricle, elastic cartilage can be found in the head and neck region as a basic component of the external ear canal, nose and epiglottis and usually does not subjected to calcification or ossification changes. Nevertheless, such changes rarely can occur in the auricular cartilage and have been described by various terms like dystrophic or metastatic calcification and heterotopic or ectopic ossification [16].

Calcification consisted of calcium deposition in a damaged tissue while calcium and phosphate levels are within normal limits in serum. Dystrophic calcification can resulted from mechanical trauma and frostbite, while metastatic calcification occurs in hypercalcemia, milk-alkali syndrome, vitamin D intoxication, hyperparathyroidism and sarcoidosis [16,20].

Ossification involves new bone development histologically resembling trabecular bone. Ectopic ossification of the auricle (auricular occificans) involves bone formation by the deposition of calcium and phosphorus in a proteinaceous matrix as hydroxyapatite crystals in a tissue that normally does not ossify [4]. X-ray usually demonstrates opacity similar to that of a normal bone [19].

In general, ectopic ossification is considered as primary when it occurs de novo and secondary if it develops in a pre-existing lesion. Congenital plaquelike osteomatosis, Albright hereditary osteodystrophy, fibrodysplasia ossificans progressiva and osseous heteroplasia can cause primary ectopic ossification in the human body [10]. Various traumatic, inflammatory or neoplastic conditions can cause secondary ossification, as well as, lesions like pilomatricoma, benign melanocytic nevi, chondroid syringoma (mixed tumor) or acne scarring [23]. Furthermore, collagen vascular disease, such as scleroderma, CREST syndrome morphea and childhood dermatomyositis may also demonstrate, except of cutaneous calcification, areas with ectopic ossification [24].

Clinical manifestations of these conditions are various. The majority of the patients are asymptomatic and the auricle does not change its configuration, thus the physician is rarely consulted. Lister [15] in his review noted that the majority of the patients were male more than 50 yrs-old. Only 4 out of 66 patients had symptoms attributed directly to auricular changes. Thus, some patients complained for discomfort when pressure applied in their external ear, i.e. wearing a cap over the head or during resting, while others reported decreased sensation of the auricle.

The rigidity is progressive over time, as depicted in our case, and usually bilateral involvement is more frequent than the unilateral one. The findings of the physical examination are typical of a partially or complete stiffness of the external ear sparing the earlobe, usually without visible cutaneous irregularity. The diagnosis can easily be made by palpation and patient's clinical history.

CT scan can easily depict the hyperdense areas along the auricles, as it is a very sensitive method for the detection of calcifications/ossifications. Furthermore, the detailed scanning with reconstruction and coronal, sagittal and 3D reformatted images can easily demonstrate the size of the abnormal areas. On top of that, the presence of radiolucent areas within the dense opacities is a very strong indicative factor for ossification and true bone formation along the auricles, and can differentiate calcification from ossification [9] (Figures 2a, b, c), thus offering valuable information for a further diagnostic work-up.

Laboratory evaluation is also helpful for detecting any possible metabolic or endocrine underlying cause of this condition. Ossification and calcification of the auricle is clinically identical, thus differentiation between them and confirmation of the diagnosis should be done by histology, although as we mentioned computed tomography studies can also be indicative of ossification demonstrating a trabecular pattern of the ossified auricle [9].

Both conditions may remain localized or diffusely involved the auricle, sometimes with extension to the external ear canal cartilage. Local ossification or calcification often results from local injury, like that due to frostbite or mechanical trauma and clinically presented as a circumscribed, unilateral lesion usually in the upper, outer rim of the auricular helix. On the contrary metabolic and endocrine diseases usually result in bilateral, symmetrical and more diffuse patterns of ossification or calcification.

Considering the incidence of ossification or calcification of the external ear in the general population, Scherrer in his large series examined 800 patients, aged 15 to 75 years, but he did not found anyone with auricular rigidity [17]. Gordon stated that 3% had radiological evidence of calcification of the auricle, however each one of these patients suffered from diseases known as causative factors to ectopic calcification such as, scleroderma, acromegaly, and diabetes mellitus [16].

Many conditions have been reported over time as causative factors in the development of ossified or calcified auricles. Severe hypothermia has been considered as the most common cause of auricular ossificans [4]. Rapid cooling has been suggested that can produce vascular thrombosis and occlusion and consequently the resulting ischemia can induce lamellar bone proliferation [25]. There have also been reported cases where ossification or calcification occurred secondary to recurrent cold exposure without frostbite [11] or even after consistent auricle manipulation [13]. Bochdalek [1] was the first suggested that ectopic bone formation might take place in reaction to bone morphogenetic protein that is released in response to certain injury or damage. Since then many authors have described rigid auricles due to local injury like mechanical trauma [12,16,17], insect bites [17], or radiation therapy [20]. Various inflammatory conditions have also been implicated as causative factors like chondritis [17], perichondritis [16-18], and syphilitic perichondritis [16,17,22]. Various systemic diseases, i.e. metabolic and endocrine disorders have been implicated with ossification or calcification of various cartilaginous structures in the human body, however auricular involvement rarely reported. Addison's disease is the most common endocrinopathy associated with this condition [16,26,27], while hypopituitarism, diabetes mellitus, acromegaly and hypothyroidism have also been reported [21,22]. There are some systemic diseases like hypertension [2], scleroderma and polyarteritis nodosa [8], systemic condromalacia [28], alkaptonuria [2] and familial cold hypersensitivity [16], which have been associated with calcification but not true ossification of the external ear. However, the pathophysiologic mechanism of either calcification or ossification in all of the aforementioned conditions has not been fully understood till now.

In our patient, Addison's disease and diabetes mellitus could be considered among the possible causative factors for his bilateral auricular ossification. Table 1 summarizes the demographic data of 18 cases including ours, with histologically documented auricular ossification.

**Table 1:** Data of 18 documented cases of auricular ossification

**Author (y)**	Age (yrs)/sex*	**Bilateral/Unilateral**^†^	**Cause**
Current case (2009)	73/M	R and L	Addison's disease and diabetes mellitus

Carfrae - Foyt (2008)[5]	49/M	L	Unknown

Sterneberg-Vos et al (2007) [6]	70/M	Unilateral	Frostbite

Gonzalez-Sixton et al (2006) [7]	65/M	R and L	Hypothermia

Manni et al (2005) [8]	63/F	R and L	Unknown

High et al (2004) [9]	60/M	R and L	Unknown

Stites et al (2003) [1]	65/M	L	Cold injury

Yeatman and Varigos (1998) [10]	66/M	R	Cold injury

Lautenschlager et al (1994) [11]	66/M	R and L	Recurrent cold injury

Cohen et al (1991) [12]	46/M	R and L	Addison's disease

Lari et al (1989) [13]	17/M	R and L	Trauma

Cohen et al (1989) [14]	70/M	R and L	Addison's disease

DiBartolomeo (1985) [4]	77/M	R and L	Cold injury

	72/M	R and L	Cold injury

Lister (1969) [15]	58/M	R and L	Unknown

Gordon (1964) [16]	34/F	R and L	Perichondritis

Scherrer (1932) [17]	53/F	R and L	Unknown

Knapp (1890) [18]	24/M	Unilateral	Perichondritis

• M: male, F: female, ^† ^R: right, L: left.

There is not a specific treatment for this unusual, irreversible condition. The fact that is a rare condition and usually asymptomatic limits the information about its treatment. When the patient complaints for serious discomfort with pain, making sleeping very difficult, one should consider surgical intervention for symptoms relief. Lister reported a case in which performed wedge biopsy of a calcified auricle curing that way patient's insomnia [15]. Lari et al [13], performed conchal reduction through a posterior incision for treatment of this condition, while Manni and Berenos-Riley [8] reported a case in which there was necessity of surgical resection of the ossified cartilage external ear canal and tragus for patient's relief. Our patient's complaint was only a mild discomfort when sleeping, thus he denied any major surgical intervention in his auricles, except from an excisional biopsy for documentation of ossification. Besides, because of the small number of reporting cases and the unclear pathogenetic mechanism of this condition, there is limited experience for the proper treatment modalities.

## Conclusion

Conclusively, true auricular ossification although is a very rare condition, requires thorough evaluation from the physician, as there is a possibility of a co-existed serious diseases like endocrinopathy. The contribution of computed tomography, could be of great importance, as not only can easily depict the abnormality but can also be strongly indicative of true ossification.

## Consent

Written informed consent was obtained from the patient for publication of this case report and accompanying images. A copy of the written consent is available for review by the Editor-in-Chief of this journal.

## Competing interests

The authors declare that they have no competing interests.

## Authors' contributions

NSM conceived and wrote the study, PZ and CK did the radiological evaluation, VS was the internist referred the patient to us and contribute the literature review, EG did the histological evaluation and TS and PDG along with NSM, jointly prepared the final manuscript
